# Genetic evidence for causal relationships between brain functional networks and domain-specific recovery after nondisabling ischemic stroke

**DOI:** 10.3389/ebm.2026.10948

**Published:** 2026-07-13

**Authors:** Huan Cai, Zhenchun Huang, Jialin Liang, Hao Zhang, Zhonghua Liu

**Affiliations:** 1 Department of Rehabilitation, Zhongshan City People’s Hospital, Zhongshan, Guangdong, China; 2 Department of Cardiology, Shantou Central Hospital, Shantou, Guangdong, China; 3 Department of Endocrinology and Metabolism, Zhongshan City People’s Hospital, Zhongshan, Guangdong, China; 4 Department of Neurology, Affiliated Hangzhou First People’s Hospital, Westlake University School of Medicine, Hangzhou, Zhejiang, China

**Keywords:** functional connectivity, lipid metabolism, Mendelian randomization, rs-fMRI, stroke recovery

## Abstract

Intrinsic brain networks are crucial for post-stroke recovery, but the causal relationships between specific networks and domain-specific recovery outcomes, as well as the role of lipid metabolism, remain unclear. This study leveraged Mendelian randomization (MR) to evaluate 191 resting-state functional magnetic resonance imaging (rs-fMRI) BOLD-derived phenotypes in relation to post-stroke recovery after nondisabling ischemic stroke. Genetic instruments for rs-fMRI phenotypes were derived from a UK Biobank genome-wide association study (n = 34,691). Outcomes included motor, cognitive, and global recovery after nondisabling ischemic stroke, assessed using longitudinal National Institutes of Health Stroke Scale subscales over 2 years (n = 1,270). Primary analyses used the multiplicative random-effects inverse-variance weighted method. A two-step MR analysis investigated whether brain networks mediate the effects of lipids on post-stroke outcomes. Higher BOLD-derived functional connectivity within the triple network (default mode network, central executive network, and salience network) was associated with better motor and cognitive outcomes. Higher genetically predicted orbitofrontal node amplitude in the limbic network correlated with better motor recovery, while stronger parieto-frontal connectivity was associated with cognitive recovery. Genetically proxied higher low-density lipoprotein cholesterol (LDL-C) was associated with poorer cognitive recovery, with evidence suggesting partial mediation through differences in BOLD-derived triple-network connectivity. This MR study supports a potential causal role of BOLD-derived functional network phenotypes, particularly the triple network, in motor and cognitive recovery, and further suggests that differences in triple-network connectivity act as a partial mediator linking elevated LDL-C liability to impaired cognitive recovery. These findings provide hypothesis-generating evidence for future mechanistic studies and for exploring whether specific brain network-targeted interventions could have a role in stroke recovery.

## Impact statement

While correlations between functional connectivity and specific aspects of stroke recovery have been reported, the causal nature of these associations warrants clarification. This study provides genetic evidence supporting potential causal contributions of specific intrinsic brain networks in post-stroke recovery, moving beyond prior correlational findings. It identifies genetically predicted higher BOLD-derived functional connectivity within the triple network as relevant to both motor and cognitive recovery and suggests that elevated low-density lipoprotein cholesterol was associated with poorer cognitive outcomes partially through differences in this network. These findings offer a preliminary and hypothesis generating rationale for targeting triple network connectivity as a potential biomarker and candidate intervention-relevant network feature in post-stroke recovery, and for testing whether lipid management is associated with preservation of intrinsic network function and cognitive recovery in prospective clinical studies.

## Introduction

Ischemic stroke is a leading cause of death and disability worldwide, highlighting the need to understand its risk factors and post-stroke recovery mechanisms. Despite advances in understanding stroke pathogenesis, the mechanisms driving post-stroke functional recovery remain poorly understood [1].

Functional connectivity (FC) refers to temporal dependencies in neural activity across distinct brain regions and is fundamental to neural plasticity [2]. Resting-state functional magnetic resonance imaging (rs-fMRI) non-invasively maps these connectivity patterns by measuring spontaneous low-frequency blood oxygen level–dependent (BOLD) signal fluctuations, which indirectly index neural activity through the hemodynamic response and are influenced by cerebral blood flow, vascular reactivity, oxygen metabolism, and endothelial function [2]. Given the importance of neural plasticity in post-stroke recovery, FC was prioritized as a key network-level phenotype for investigating the brain’s capacity to reorganize and support recovery after stroke-related injury.

Growing evidence links altered intrinsic brain functional networks to post-stroke recovery outcomes. Disrupted FC after a stroke correlates with functional impairment, while its reestablishment facilitates recovery [3–5]. Although FC correlates with specific aspects of stroke recovery [6], such as motor function [7, 8] and cognition [9], causality remains unclear.

Mendelian randomization (MR) infers causality using genetic variants as instrumental variables (IVs). A recent bidirectional MR study did not establish robust causal links between 191 rs-fMRI phenotypes and post-stroke functional recovery [10], potentially attributable to the use of the modified Rankin Scale (mRS), which combines varied neurological impairments into a single measure and may obscure domain-specific recovery. No MR study has yet examined causal relationships between FC and domain-specific stroke recovery.

Furthermore, emerging evidence implicates a complex interplay between lipid profiles and brain networks. Imbalances in lipid metabolism may disrupt FC, especially in regions associated with cognition [11, 12], which are critical for post-stroke recovery. However, whether brain networks can mediate the effect of blood lipids on recovery remains unknown.

Thus, this study employed MR to evaluate relationships between intrinsic rs-fMRI phenotypes and domain-specific stroke recovery, as well as whether these network phenotypes mediate associations between lifelong genetic liability to lipid traits and stroke recovery.

## Materials and methods

**FIGURE 1:**
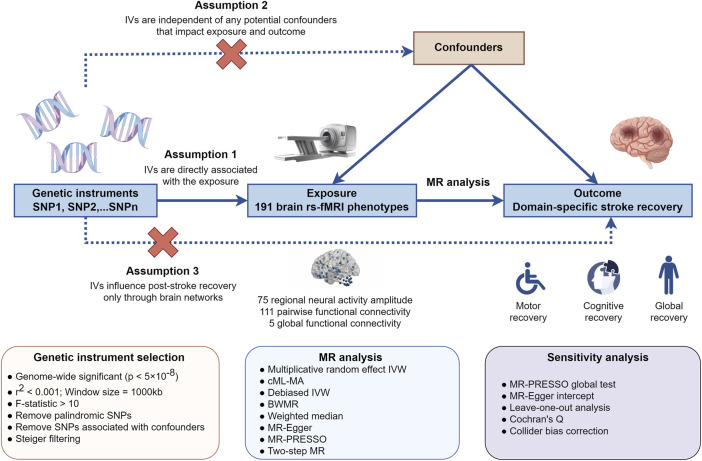
Study design and three core assumptions of the MR analysis. IV, instrumental variable; MR, Mendelian randomization; SNP, single-nucleotide polymorphism; rs-fMRI, resting-state functional magnetic resonance imaging; IVW, inverse-variance weighted; cML-MA, Constrained Maximum Likelihood Mixed-Model Association; MR-PRESSO, MR-Pleiotropy Residual Sum and Outlier; BWMR, Bayesian Weighted MR.

All summary-level genomic data are publicly available (Supplementary Table 1 in the electronic Supplementary Material). The study design is shown in Figure 1. All data sources were approved by relevant institutional review boards in the original studies, and all participants provided informed consent. This study follows the STROBE-MR statement [13].

### Genome-wide association study (GWAS) of 191 rs-fMRI phenotypes

Summary GWAS data for rs-fMRI phenotypes were sourced from the UK Biobank (n = 34,691) [14]. Fluctuation amplitude was defined as the temporal standard deviation of node features within a given component. Pairwise FC was defined as temporal correlation between BOLD signal time series for each pair of nodes. Global connectivity was defined by extracting the top six principal components from the node adjacency matrix. At the genome-wide significance level, 191 phenotypes were linked to genetic variation, comprising 75 regional BOLD fluctuation amplitude traits, 111 pairwise FC traits, and 5 global FC traits (Supplementary Table 2).

### GWAS of domain-specific stroke recovery

Genetic associations for post-stroke motor, cognitive, and global recovery were obtained from a GWAS of 1,270 nondisabling ischemic stroke patients, defined as modified Rankin Scale (mRS) ≤ 3, from the Vitamin Intervention for Stroke Prevention (VISP) trial with a National Institutes of Health Stroke Scale (NIHSS) score greater than zero at randomization and no recurrent stroke [15]. All participants were followed up every 3 months for 2 years.

Three recovery phenotypes were defined by changes in motor, cognitive, and global impairments at each time point. Motor impairment was measured as the change in the sum of the NIHSS motor scores for Facial Palsy, Dysarthria, Motor Arm, and Motor Leg, termed NIH Motor-6. Similarly, cognitive impairment was defined as the change in the sum of the NIHSS cognitive scores, termed NIHSS Cog-4, for Level of Consciousness (LOC) Questions, LOC Commands, Aphasia, and Inattention/Neglect. Global impairment was the change in the total NIHSS score. A linear mixed-effects model estimated the changes in NIHSS scores for each phenotype (delta = initial score − follow-up score) [15].

### Genetic instrument selection

We first selected independent single-nucleotide polymorphisms (SNPs) (*r* ^*2*^ < 0.001, window size = 1,000 kb) with genome-wide significance (*p* < 5 × 10^−8^). SNPs with an *F*-statistic < 10 were excluded. We then excluded SNPs linked to confounders (e.g., depression [16], migraine [17], insomnia [16]) affecting both brain networks and post-stroke functional outcomes [18–20]. We used the GWAS Catalog database to identify and exclude these SNPs (Supplementary Table 3). Third, we applied Steiger filtering to reduce the likelihood of reverse-direction instruments, and removed networks with fewer than three IVs required for some sensitivity analyses.

Negligible sample overlap between exposure and outcome datasets was confirmed. We used proxy SNPs (*r* ^*2*^ > 0.8) from 1000 Genomes European reference data if needed, and removed ambiguous palindromic SNPs.

### Two-sample MR analyses

Multiplicative random-effects inverse-variance weighted (IVW) MR was the primary method. Supplementary analyses included weighted median [21], MR-Egger [22], Constrained Maximum Likelihood Mixed-Model Association [23], debiased IVW [24], and Bayesian Weighted Mendelian Randomization [25]. MR-Pleiotropy Residual Sum and Outlier (MR-PRESSO) was used to identify potential outliers [26]. These methods collectively enhance robustness to IV assumption violations.

A causal relationship was inferred if all MR methods showed directionally consistent estimates and the IVW *p* < 8.73 × 10^−5^ (Bonferroni correction for 191 phenotypes and 3 outcomes).

### Sensitivity analyses

We tested SNP heterogeneity with Cochran’s *Q* statistic and used leave-one-out plots to check if single SNPs drove the results. In addition, we repeated our analysis using a relaxed IV selection threshold of *p* < 1 × 10^−6^ to check for consistency in the direction with those obtained from the genome-wide significant threshold. To assess the possibility of reverse causality, we performed reverse MR analyses in significant rs-fMRI phenotypes from forward MR using IVs with a suggestive significance threshold (*p* < 5 × 10^−6^). MR-PRESSO global test and MR-Egger intercept were used to detect horizontal pleiotropy.

To address collider bias risk when analyses are conditioned on ischemic stroke survivors, we repeated MR analyses using genetic effects on recovery outcomes adjusted via corrected weighted least squares (CWLS) [27], based on summary ischemic stroke data from the GIGASTROKE consortium of European ancestry [28].

### Mediation MR

We used two-step MR to examine whether genetically proxied lipid traits were linked to post-stroke outcomes through BOLD-derived rs-fMRI network phenotypes. Summary statistics of three blood lipid measures, including triglycerides, low-density lipoprotein cholesterol (LDL-C), and high-density lipoprotein cholesterol (HDL-C), were obtained from the most recent circulating metabolite GWAS analysis of 136,016 participants [29]. We first estimated the causal effect of lipid traits on intrinsic functional networks (coefficient β_XM_) and on post-stroke recovery patterns (coefficient β_XY_), then assessed the causal effect of rs-fMRI phenotypes on specific subdomains of post-ischemic stroke recovery (coefficient β_MY_). We calculated the mediation effect of BOLD-derived network phenotypes using the product of coefficients method [30]. The mediation effect (β_XM_ × β_MY_) was then divided by the total effect (β_XY_) to calculate the proportion of the mediation effect. Standard errors for the mediation effect were calculated with the delta method. Notably, only rs-fMRI phenotypes showing robust evidence in the primary univariable MR were taken forward to mediation analyses.

Lipid and rs-fMRI traits were measured continuously and standardized to standard deviations. Analyses were performed in R (v4.2.1) with TwoSampleMR (v0.6.8) [31], RMediation (v1.2.2) [32], and MRPRESSO (v1.0) [26] packages.

## Results

### Genetically predicted rs-fMRI phenotypes and specific domains of post-stroke recovery

**FIGURE 2:**
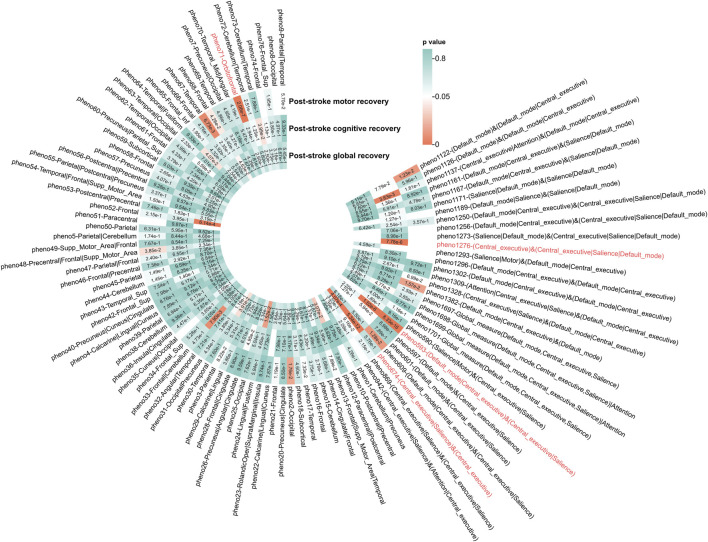
Circular heatmap for the IVW MR estimates of associations between rs-fMRI phenotypes and domain-specific stroke recovery.

**FIGURE 3:**
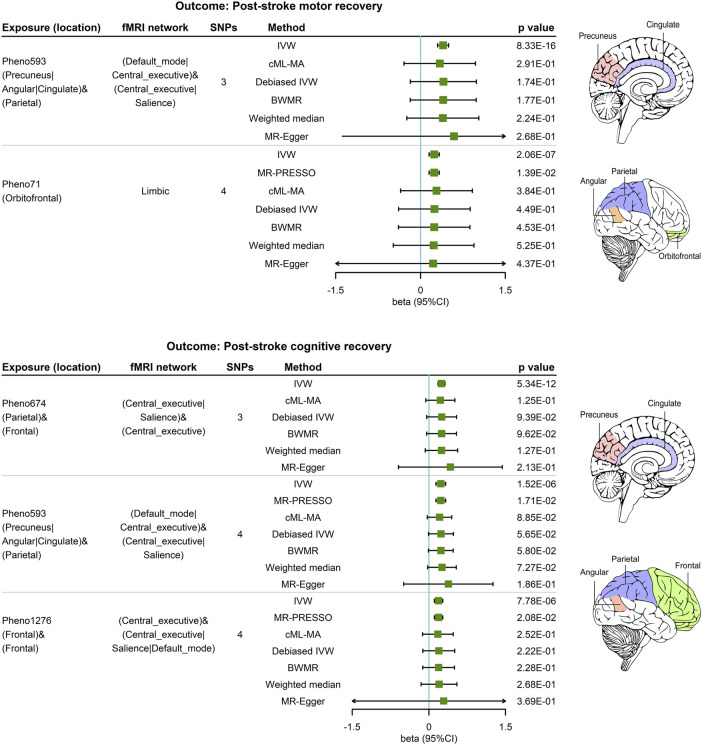
Causal relationships that remained significant after Bonferroni correction in MR analysis results. Left: forest plot illustrating the significant causal estimates using six MR methods. Right: pattern diagram showing the brain anatomical regions of the corresponding rs-fMRI phenotypes. CI, confidence interval.

The full primary MR results are shown in Supplementary Tables 4–6. Twenty-four rs-fMRI phenotypes were nominally associated with domain-specific stroke recovery (*p* < 0.05, Figure 2). We identified four BOLD-derived brain functional network phenotypes showing robust MR evidence of association with two distinct recovery phenotypes (motor and cognitive recovery) after Bonferroni correction in the primary IVW analysis (Figure 3). The genetic variants used as instruments for significant intrinsic brain activity phenotypes are presented in Supplementary Table 7. However, no significant association survived multiple testing correction for post-stroke global recovery.

Two rs-fMRI phenotypes showed genetically supported evidence of association with motor improvement (Figure 3). Pheno593 reflects connectivity in the precuneus, angular gyrus, or cingulate, and parietal regions. The BOLD-derived signals of these brain regions involve FC in the triple network (default mode network (DMN)/central executive network (CEN) and CEN/salience network (SN)). Higher genetically predicted FC in these networks was associated with greater motor improvement (IVW β = 0.399, 95% confidence interval [CI] = 0.302–0.496, *p* = 8.33 × 10^−16^). In the limbic network, increased BOLD fluctuation amplitude of the orbitofrontal region was linked to a better motor outcome (IVW β = 0.241, 95% CI = 0.150–0.331, *p* = 7.78 × 10^−6^).

Three rs-fMRI phenotypes showed evidence for a potential causal contribution to post-stroke cognitive recovery (Figure 3). Pheno674 involves FC between parietal and frontal regions. Higher genetically predicted CEN-SN connectivity was associated with a better cognitive outcome (IVW β = 0.245, 95% CI = 0.176–0.315, *p* = 5.34 × 10^−12^). For Pheno593, connectivity in the precuneus, angular gyrus, or cingulate area, and parietal area positively correlated with cognitive recovery (IVW β = 0.232, 95% CI = 0.138–0.327, *p* = 1.52 × 10^−6^). For Pheno1276, resting frontal BOLD-derived signal features were linked to cognitive changes after stroke, with higher FC between CEN and SN or DMN or CEN associated with greater cognitive improvement (IVW β = 0.188, 95% CI = 0.106–0.270, *p* = 7.78 × 10^−6^).

These findings were supported by other MR analyses (Figure 3; Supplementary Tables 8,9). MR-PRESSO detected no significant outliers in exposure-outcome pairs for which the test was applicable.

### Sensitivity analysis

Cochran’s *Q* test showed no statistical evidence of heterogeneity among individual SNPs (Table 1). The scatterplots, forest plots, and leave-one-out plots suggested that no single SNP drove the associations (Supplementary Figures 1–6 in the electronic Supplementary Material). No statistical evidence of horizontal pleiotropy was detected by MR-PRESSO global test and MR-Egger intercept (all *p* > 0.05, Table 1). Results using the relaxed threshold were still statistically significant and directionally consistent with the primary analysis (Supplementary Table 10). Reverse MR analyses (Supplementary Tables 11, 12) showed no evidence of reverse causality. Associations remained stable after adjusting for collider bias (Supplementary Tables 8,9).

**TABLE 1:** Pleiotropy and heterogeneity tests for significant exposure-outcome pairs in forward Mendelian randomization.

Exposure	Outcome	Pleiotropy			Heterogeneity (IVW)	
		MR-PRESSO global *p* value	MR-Egger intercept	*p* value	Cochran’s *Q*	*p* value
Pheno593	Post-stroke motor recovery	NA	−0.018	0.851	0.057	0.972
Pheno71	Post-stroke motor recovery	0.994	0.018	0.911	0.064	0.996
Pheno593	Post-stroke cognitive recovery	0.909	−0.009	0.776	0.479	0.923
Pheno1276	Post-stroke cognitive recovery	0.974	−0.008	0.876	0.226	0.973
Pheno674	Post-stroke cognitive recovery	NA	−0.022	0.794	0.120	0.942

Abbreviations: MR-PRESSO, Mendelian randomization-pleiotropy residual sum and outlier; IVW, inverse-variance weighted; NA, not applicable.

### Mediation analysis

**FIGURE 4:**
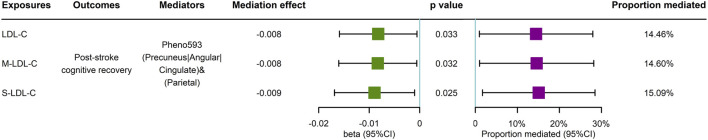
Associations between genetically predicted LDL-C, M-LDL-C, S-LDL-C and post-stroke cognitive recovery mediated by brain networks. LDL-C: low-density lipoprotein cholesterol; M-LDL-C, total cholesterol in medium LDL; S-LDL-C, total cholesterol in small LDL.

We found that genetically predicted higher LDL-C levels, but not triglycerides or HDL-C, were associated with poorer cognitive recovery (IVW β = −0.057; 95% CI = −0.105, −0.008; *p* = 0.022, Supplementary Figures 7,8). We found no MR evidence supporting associations between lipid traits and motor recovery. We then conducted MR analysis of LDL-C and its subfractions, including total cholesterol in large LDL, total cholesterol in medium LDL (M-LDL-C), and total cholesterol in small LDL (S-LDL-C), to examine whether they were associated with three imaging phenotypes that showed a potential causal contribution to post-stroke cognitive recovery. We found that LDL-C and its subfractions were associated with Pheno593, but not Pheno674 or Pheno1276 (Supplementary Figures 8,9). Finally, for Pheno593, our exploratory mediation analysis suggested that BOLD-derived connectivity in the precuneus, angular gyrus, or cingulate, and parietal regions, as well as the triple network (DMN/CEN and CEN/SN), may partially mediate the associations between genetic liability to LDL-C, M-LDL-C, and S-LDL-C and cognitive recovery (Figure 4). Specifically, the mediation effects were estimated to be −0.008 (95% CI -0.016, −0.001; *p* = 0.033) for LDL-C, −0.008 (95% CI -0.016, −0.001; *p* = 0.032) for M-LDL-C, and −0.009 (95% CI -0.017, −0.001; *p* = 0.025) for S-LDL-C, with mediation proportions of 14.46% (95% CI 0.96%–27.96%), 14.60% (95% CI 1.01%–28.18%), and 15.09% (95% CI 1.70%–28.48%), respectively. No statistical evidence of horizontal pleiotropy or heterogeneity was detected, and the Steiger directionality tests supported the inferred causal direction (Supplementary Table 13).

## Discussion

Using MR, we found that specific rs-fMRI phenotypes were associated with motor and cognitive recovery, and that BOLD-derived connectivity may partly mediate associations between genetic liability to lipid traits and cognitive recovery. These findings may help inform future research on connectivity profiles related to poor recovery.

Our results support a genetically informed association between triple-network (DMN-CEN-SN) connectivity and both motor and cognitive recovery after stroke. Higher genetically predicted BOLD-derived connectivity within this large-scale network system was associated with better recovery outcomes (Pheno593), suggesting that post-stroke recovery may depend not only on restoration of domain-specific circuits, but also on the integrity of domain-general networks that coordinate attention, executive control, self-monitoring, and adaptive learning [33, 34]. Within the triple-network framework, the SN, anchored in the anterior insula and dorsal anterior cingulate cortex, detects behaviorally relevant stimuli and facilitates dynamic switching between the DMN and the CEN. The CEN supports goal-directed control, working memory, performance monitoring, and error correction, whereas the DMN contributes to memory-based prediction, self-referential processing, and internally guided cognition [33]. These processes are directly relevant to cognitive recovery after stroke, as impairments in attention allocation, executive control, memory retrieval, and self-monitoring are common contributors to post-stroke cognitive dysfunction [33]. Consistent with this view, previous studies have shown that alterations in triple-network organization are associated with cognitive impairment [35], and that preservation or restoration of FC within and between regions of the DMN is related to better cognitive outcomes after stroke [6]. Importantly, the same network architecture may also support motor recovery. Although motor restoration is strongly influenced by the integrity of sensorimotor pathways and interhemispheric motor connectivity [6], successful motor rehabilitation also requires attention to task-relevant cues, selection of compensatory strategies, feedback-based error correction, and motor relearning [36]. These functions depend heavily on interactions between the SN and CEN and may interact with DMN-related memory and self-monitoring processes [37]. Thus, more favorable triple-network connectivity may provide a domain-general network-level scaffold through which cognitive-control systems support adaptive motor performance, particularly when primary sensorimotor pathways are disrupted [37], although the rs-fMRI phenotype may also partly index vascular support for network function.

This concurrent association with motor and cognitive recovery may reflect large-scale network integration, shared plasticity mechanisms, and vascular support for metabolically demanding recovery processes. Stroke recovery is thought to rely on experience-dependent plasticity, including unmasking of latent synaptic connections, long-term potentiation-like mechanisms, and functional reorganization of spared cortical and subcortical circuits [1]. The hubs of the SN and CEN are anatomically and functionally connected with association cortices and secondary motor regions, including the premotor cortex and supplementary motor area, through cortico-cortical and cortico-striatal-thalamic pathways [38, 39]. These pathways may serve as convergence zones where salience detection, executive control, memory-based prediction, and motor planning are integrated during rehabilitation-related learning. Therefore, stronger genetically predicted triple-network connectivity may reflect an inherited propensity for more efficient large-scale network communication or more favorable neurovascular coupling and vascular reactivity, which could facilitate plastic reorganization and coordinated recovery across both cognitive and motor domains.

Within the limbic network, we found that genetically predicted higher node amplitude in the orbitofrontal region was linked to better motor outcomes (Pheno71). The limbic network’s involvement in motor recovery may suggest an interplay between emotional/motivational factors and physical rehabilitation, expanding traditional models focused solely on sensorimotor pathways.

The parieto-frontal network is integral to various cognitive processes, including attention, working memory, and executive functions [40]. Our MR analysis showed that the parieto-frontal network (Pheno674) exhibited a positive association with better cognitive outcomes, consistent with the previous findings of reduced global and local efficiency in the parieto-frontal network in subjects with cognitive impairment [41].

In conjunction with a prior study [12], our results provide genetic evidence consistent with the possibility that FC mediates the relationship between atherogenic lipid profiles and cognition, and extend this hypothesis to stroke recovery. While LDL-C is a well-established risk factor for ischemic stroke, its role in recovery is less understood, particularly because post-stroke LDL-C levels are influenced by treatment and clinical factors. Prior research indicated that higher LDL-C levels correlated with greater cognitive impairment in patients with cerebral small vessel disease, suggesting that LDL-C may contribute to cognitive decline through vascular mechanisms [42]. Our findings suggest that genetic liability to atherogenic lipid (LDL-C, M-LDL-C, S-LDL-C) traits may be associated with poorer cognitive recovery after stroke partly through genetically proxied differences in BOLD-derived triple-network connectivity, possibly through effects on neuroinflammation [43, 44], vascular integrity, endothelial function, cerebral perfusion, neurovascular coupling, amyloidogenic factors [45], neural repair mechanisms, or shared genetic influences. Future studies should test whether optimized lipid management can preserve brain networks or mitigate cognitive deficits after stroke.

Several limitations should be considered. First, because the VISP outcome cohort consisted predominantly of patients with nondisabling ischemic stroke of European ancestry, the modest sample limits generalizability to more clinically heterogeneous populations and to other ancestry groups. Second, resting-state BOLD response reflects spontaneous low-frequency neural activity but is also influenced by neurovascular coupling, vascular reactivity, and metabolic integrity. Therefore, this phenotype may capture variation in both functional network organization and the vascular–metabolic support systems. In the context of lipid metabolism, this is particularly important because atherogenic lipid profiles may affect endothelial function, cerebral perfusion, and vascular integrity, which could in turn alter rs-fMRI connectivity estimates independently of, or in combination with, neural communication. Thus, mediation by rs-fMRI phenotypes should be interpreted as reflecting BOLD-derived neurovascular or hemodynamic pathways, not purely neural connectivity. Third, recovery phenotypes based on NIHSS subscales are relatively coarse compared to detailed assessments. Fourth, MR estimates reflect the lifelong genetic liability to BOLD-derived brain functional phenotypes, with genetic instruments for the 191 rs-fMRI traits derived from healthy UK Biobank participants. Therefore, these findings do not prove that modifying FC after stroke would improve recovery, especially given lesion-related reorganization and disconnection. Fifth, our summary-level MR design could not account for lesion localization. Lesions situated within or adjacent to the key networks examined here may themselves alter the measured FC and could modulate the observed network–recovery relationships. Because the outcome GWAS did not provide lesion-location information, we were unable to stratify or adjust for this anatomical confounder. Finally, limited sample size of the outcome GWAS and instrument availability may reduce power and precision of MR estimates, and increase susceptibility to winner’s curse or overestimation. Although Steiger directionality tests and pleiotropy sensitivity analyses supported the robustness of the mediation-related associations, shared genetic architecture and unmeasured pleiotropic pathways cannot be ruled out. The reported effect sizes for the detected associations should be interpreted as preliminary rather than definitive estimates of magnitude, especially for the mediation analysis, because the lipid instruments represent lifelong genetic predisposition rather than longitudinally measured or treated LDL-C levels. Despite these limitations, our findings suggest a biologically plausible and testable pathway linking lipid genetic liability, brain network organization, and recovery.

## Conclusion

This MR study provides preliminary genetic evidence supporting potential causal contributions of four BOLD-derived rs-fMRI phenotypes to motor and cognitive recovery after nondisabling ischemic stroke, highlighting the possible relevance of the triple network (DMN, CEN, SN). Additionally, BOLD-derived connectivity features involving DMN, CEN, and SN networks may in part mediate the association between genetically proxied lipid traits and cognitive outcomes. However, this potential pathway should be interpreted cautiously and requires validation in additional cohorts and larger GWAS datasets with more available instruments, ideally including detailed lesion localization, serial post-stroke lipid measurements, medication data, and post-stroke rs-fMRI measures. Future research could use these hypothesis-generating findings to identify candidate network targets for mechanistic studies and to evaluate whether interventions such as noninvasive brain stimulation can modulate relevant connectivity patterns and improve stroke recovery.

## Data Availability

Publicly available datasets were analyzed in this study. The summary level data for rs-fMRI phenotypes are available at https://doi.org/10.5281/zenodo.5775047. The summary level data for post-stroke recovery phenotypes and GIGASTROKE data are available at the Cerebrovascular Disease Knowledge Portal. The summary level data for blood lipid measures are available through the NHGRI-EBI GWAS Catalogue.
