# Supplementary material for: Genetic evidence for causal relationships between brain functional networks and domain-specific recovery after nondisabling ischemic stroke
Source: Exp Biol Med (Maywood). 2026 Jul 13;251:10948. doi: 10.3389/ebm.2026.10948 (PMC13402224; doi:10.3389/ebm.2026.10948)
Supplement: Supplementary file 1 [file Supplementaryfile1.docx]

**SUPPLEMENTARY**

**Genetic evidence for causal relationships between brain functional networks and domain-specific recovery after nondisabling ischemic stroke**

**Authors and Affiliations**

Huan Cai^1*^, Zhenchun Huang^2*^, Jialin Liang^3^, Hao Zhang^4^, Zhonghua Liu^1^

^1^ Department of Rehabilitation, Zhongshan City People's Hospital, Zhongshan 528403, Guangdong, China.

^2^ Department of Cardiology, Shantou Central Hospital, Shantou 515031, Guangdong, China.

^3^ Department of Endocrinology and Metabolism, Zhongshan City People's Hospital, Zhongshan 528403, Guangdong, China.

^4^ Department of Neurology, Affiliated Hangzhou First People's Hospital, Westlake University School of Medicine, Hangzhou 310006, Zhejiang, China.

**Correspondence to:**

Hao Zhang

Department of Neurology, Affiliated Hangzhou First People's Hospital, Westlake University School of Medicine, No.261, Huansha Road, Hangzhou 310006, Zhejiang, China.

Email: zh_hao_neurol@163.com

Zhonghua Liu

Department of Rehabilitation, Zhongshan City People's Hospital, No.2, East Sunwen Road, Zhongshan 528403, Guangdong, China.

Email: zhonghua_reha@163.com

^*^ These authors contributed equally to this work.


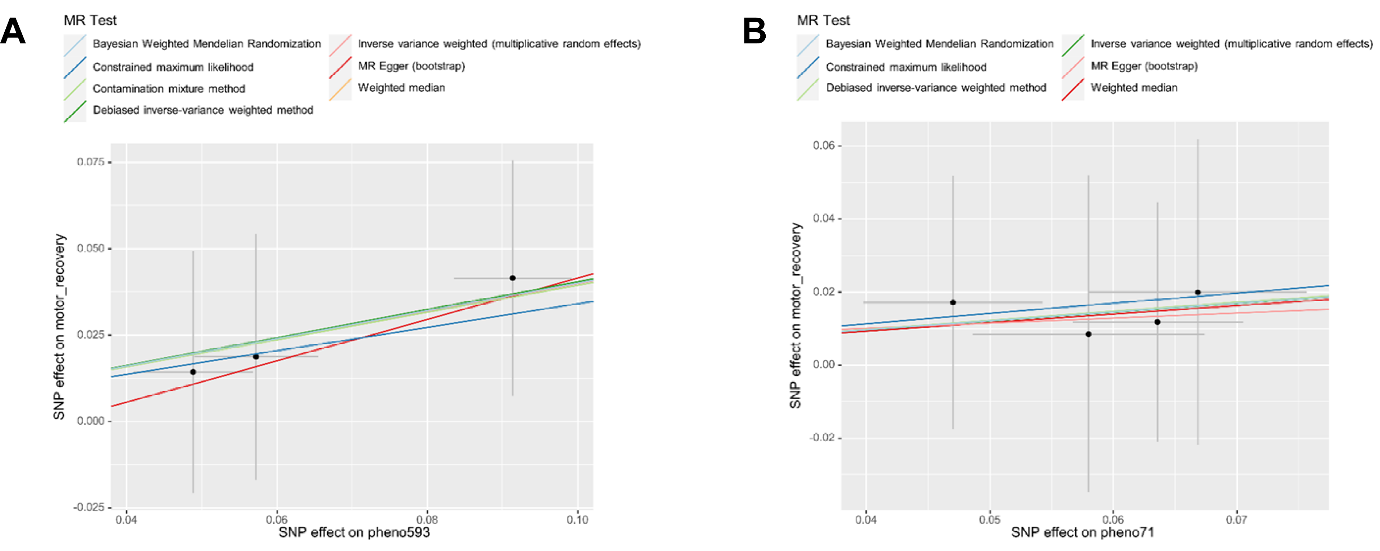


**Supplementary Figure 1** The scatter plots of the significant causal relationships between intrinsic brain activity phenotypes SNPs and motor recovery after ischemic stroke.


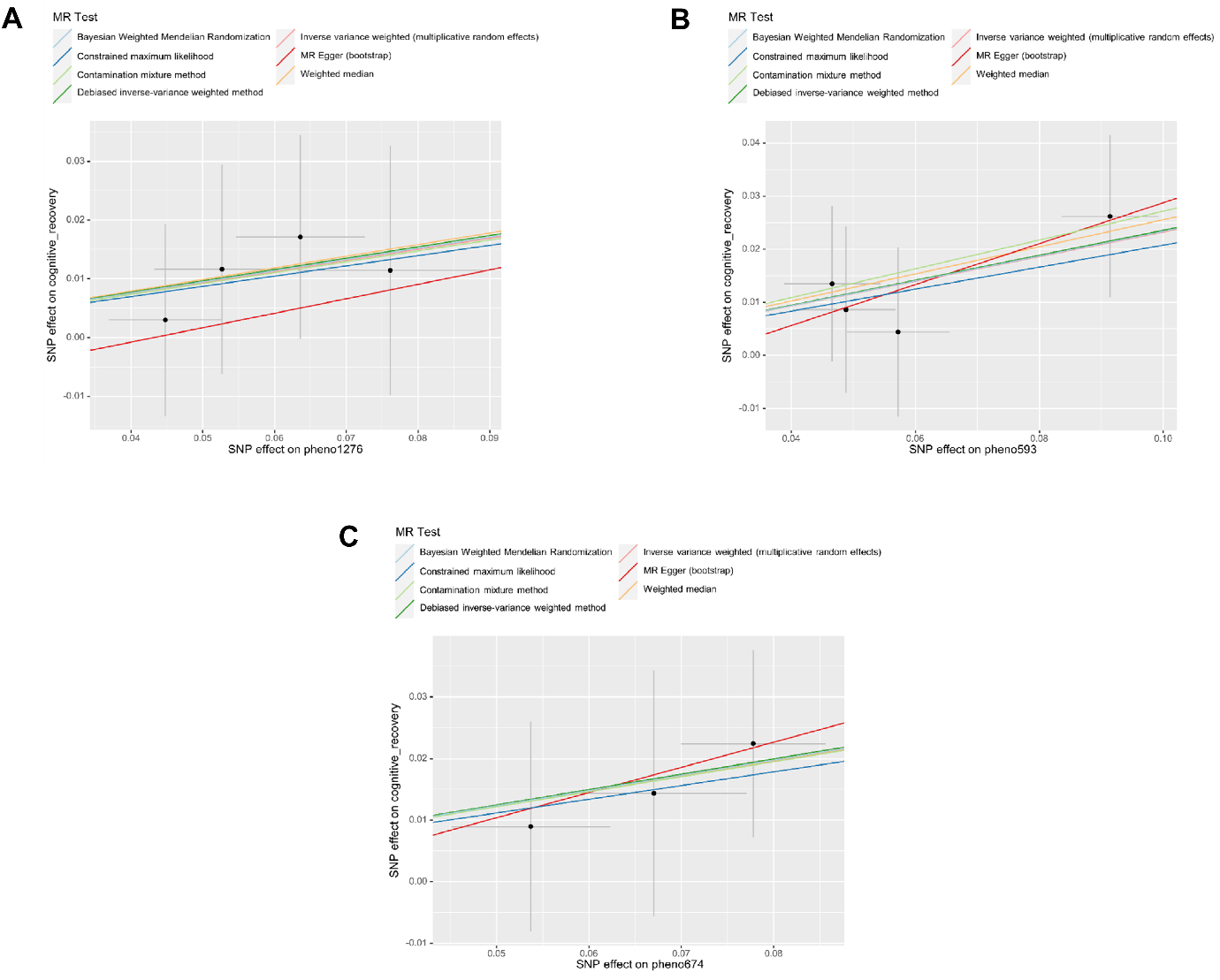


**Supplementary Figure 2** The scatter plots of the significant causal relationships between intrinsic brain activity phenotypes SNPs and cognitive recovery after ischemic stroke.


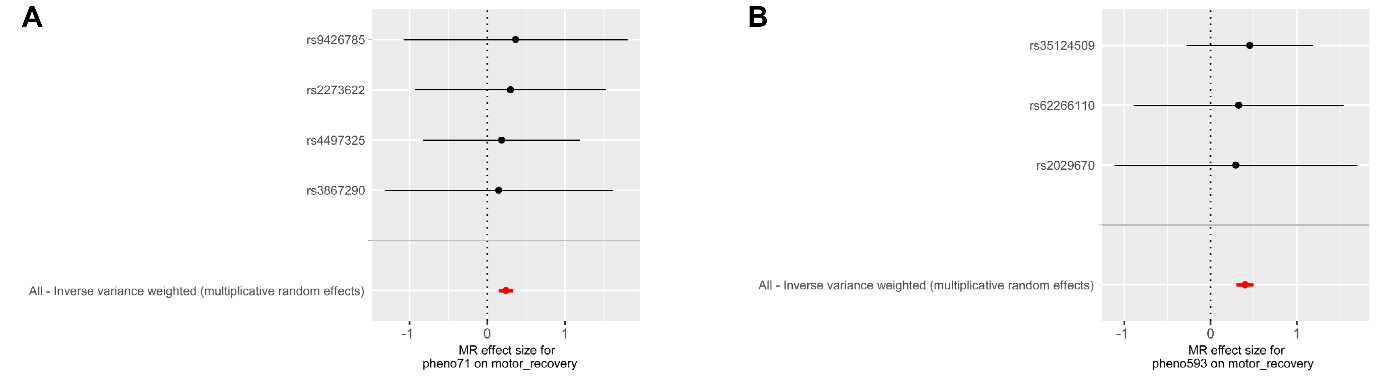


**Supplementary Figure 3** Forest plots of individual and combined SNP MR-estimated effects sizes for significant intrinsic brain activity phenotypes SNPs on motor recovery after ischemic stroke. The results were derived from the multiplicative random-effects inverse variance weighted method. Data are displayed as regression coefficient (β) and 95% confidence interval.


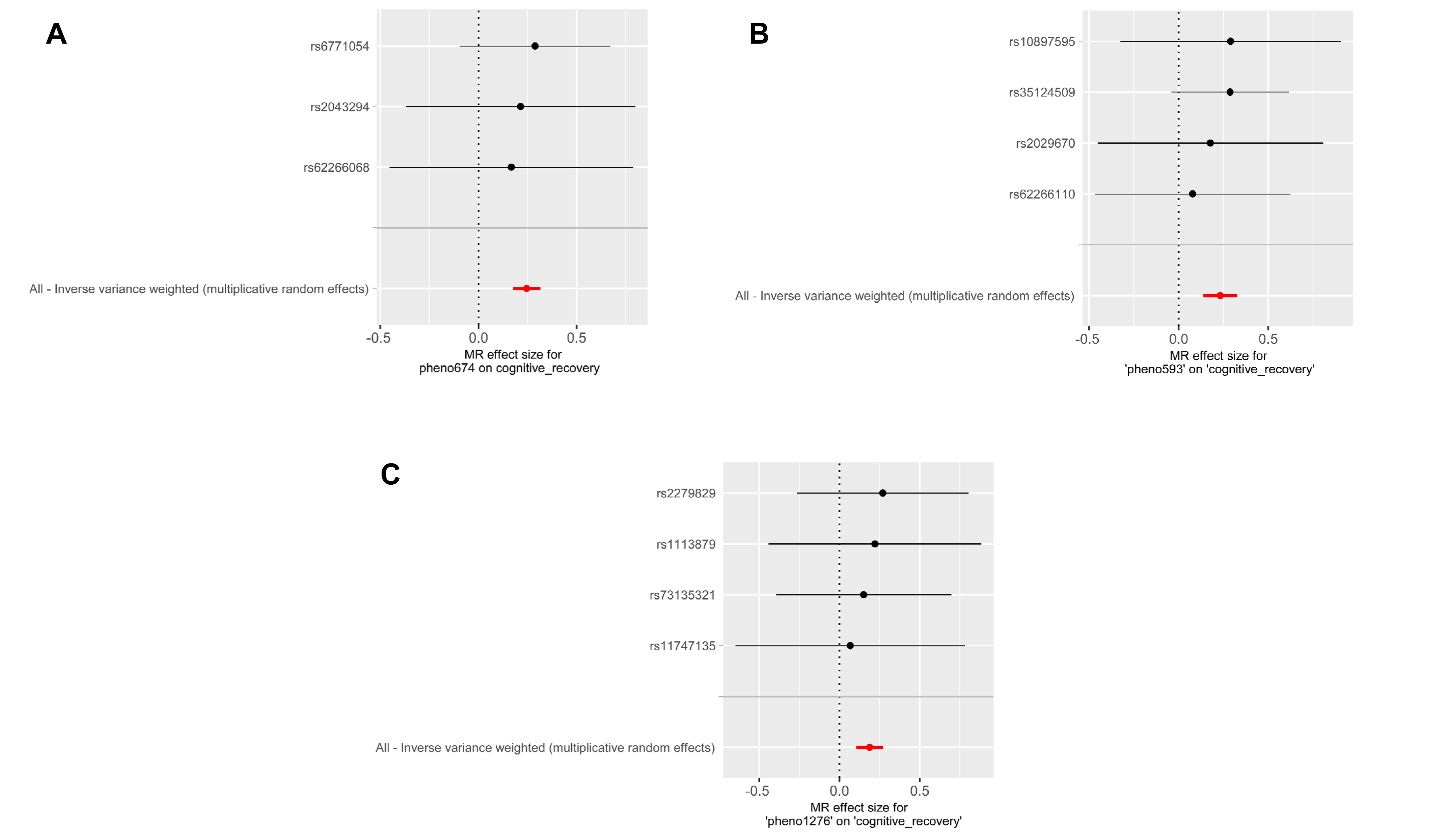


**Supplementary Figure 4** Forest plots of individual and combined SNP MR-estimated effects sizes for significant intrinsic brain activity phenotypes SNPs on cognitive recovery after ischemic stroke. The results were derived from the multiplicative random-effects inverse variance weighted method. Data are displayed as regression coefficient (β) and 95% confidence interval.


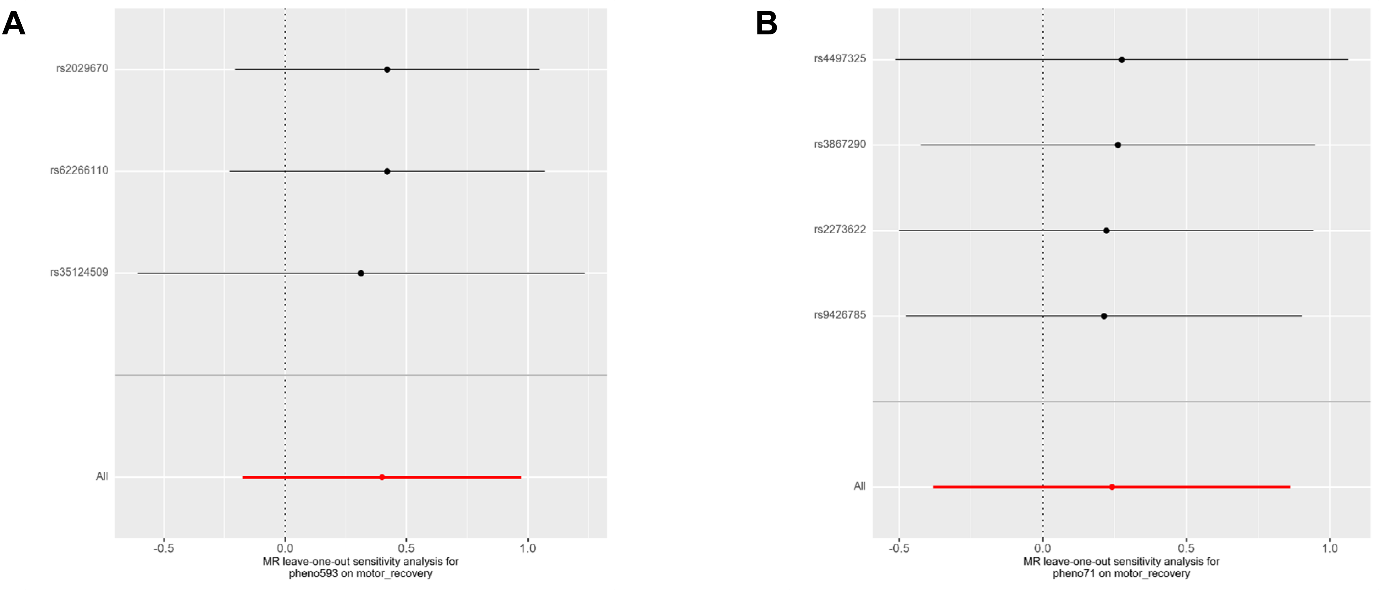


**Supplementary Figure 5** Leave-one-out analysis related to the significant intrinsic brain activity phenotypes genetic instrument with respect to motor recovery after ischemic stroke. Leave-one-out plots report the MR estimate excluding one variant at a time from the genetic instrument.


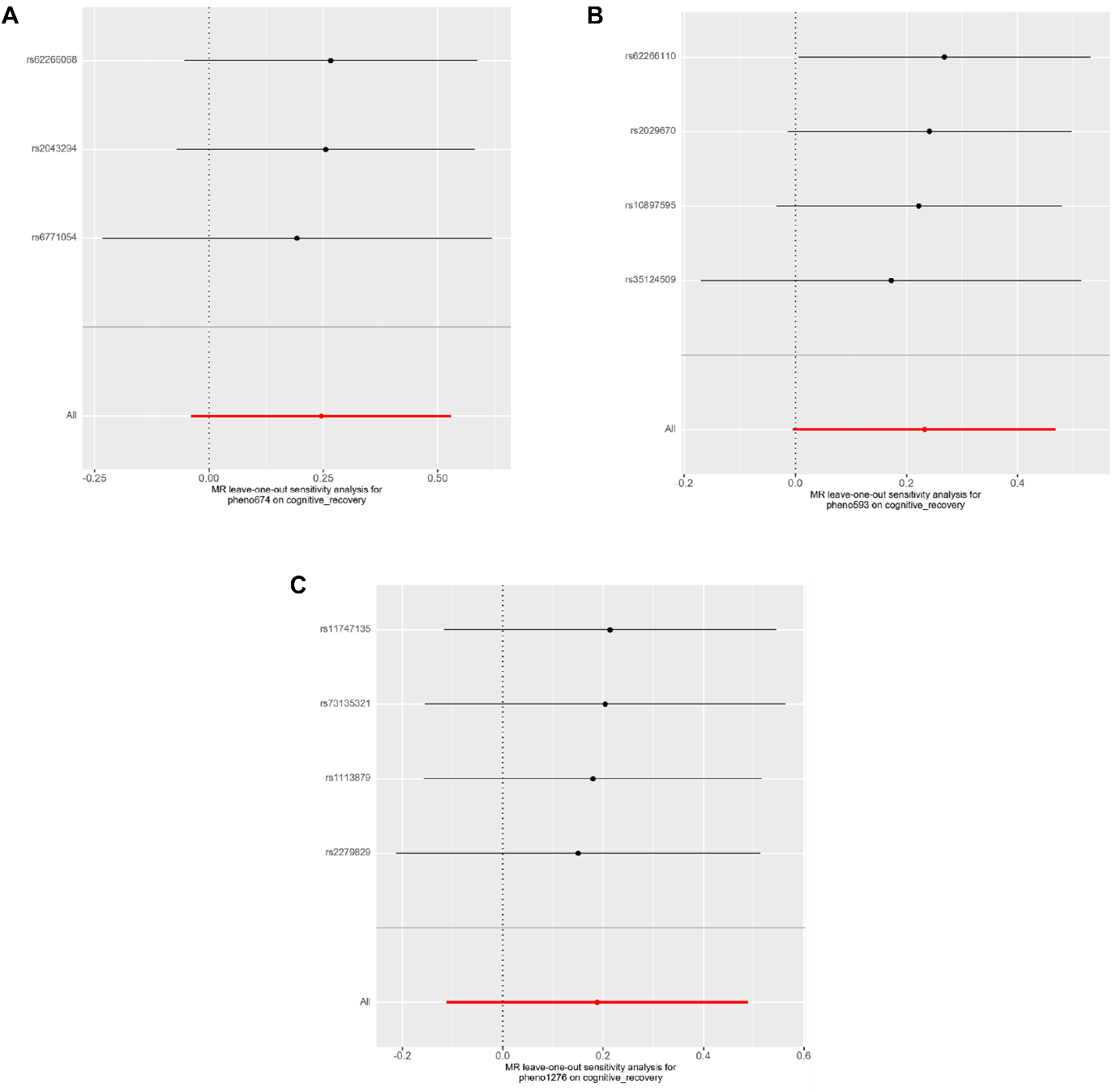


**Supplementary Figure 6** Leave-one-out analysis related to the significant intrinsic brain activity phenotypes genetic instrument with respect to cognitive recovery after ischemic stroke. Leave-one-out plots report the MR estimate excluding one variant at a time from the genetic instrument.


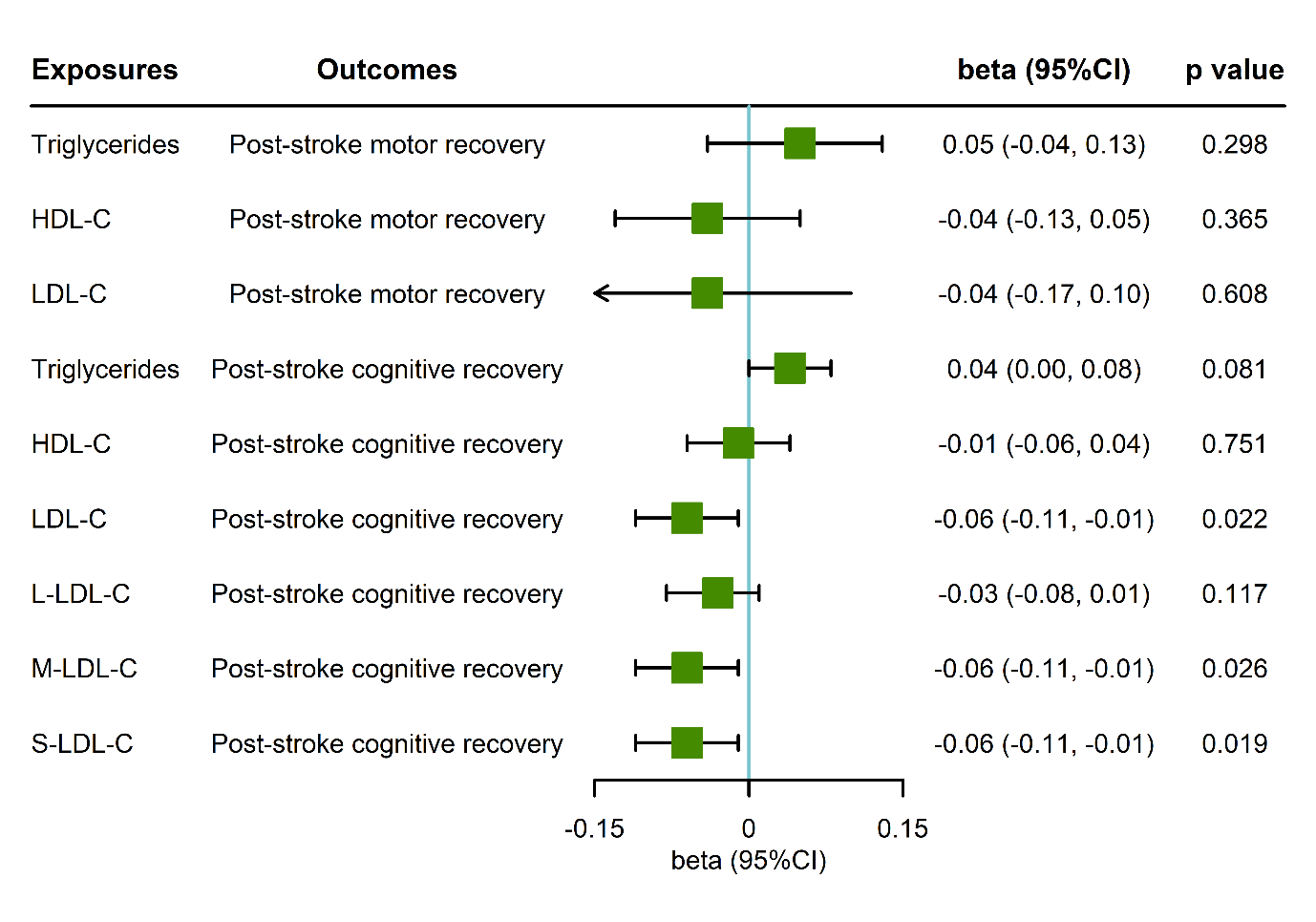
 **Supplementary Figure 7** MR associations of genetically predicted lipid traits with post-stroke recovery phenotypes; results derived from multiplicative random-effects inverse variance weighted analyses. HDL-C: high-density lipoprotein cholesterol; LDL-C: low-density lipoprotein cholesterol; L-LDL-C, total cholesterol in large LDL; M-LDL-C, total cholesterol in medium LDL; S-LDL-C, total cholesterol in small LDL.


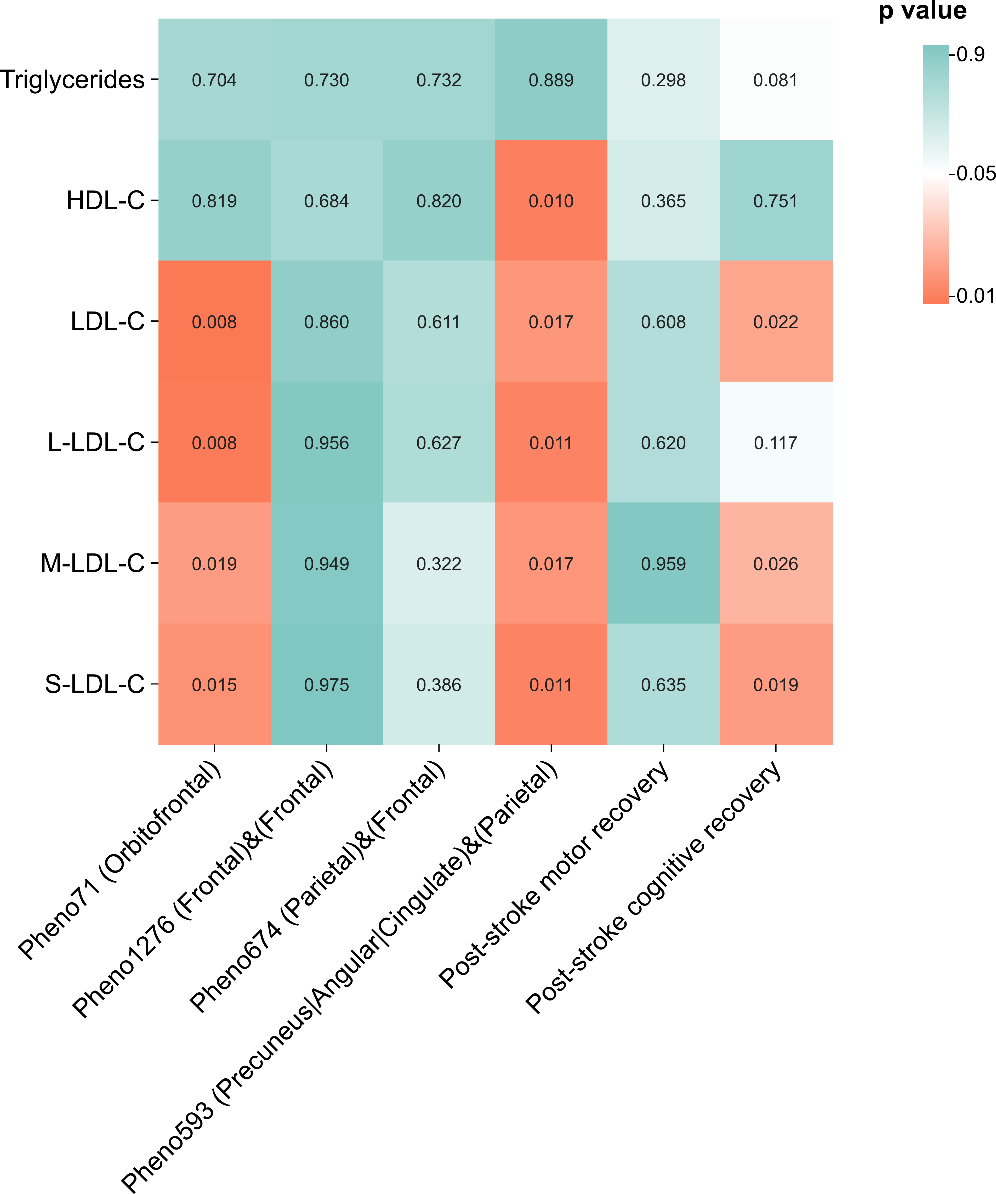


**Supplementary Figure 8** Heat map of associations of lipid traits with significant rsfMRI phenotypes and post-stroke outcomes, which was presented by *p* value.


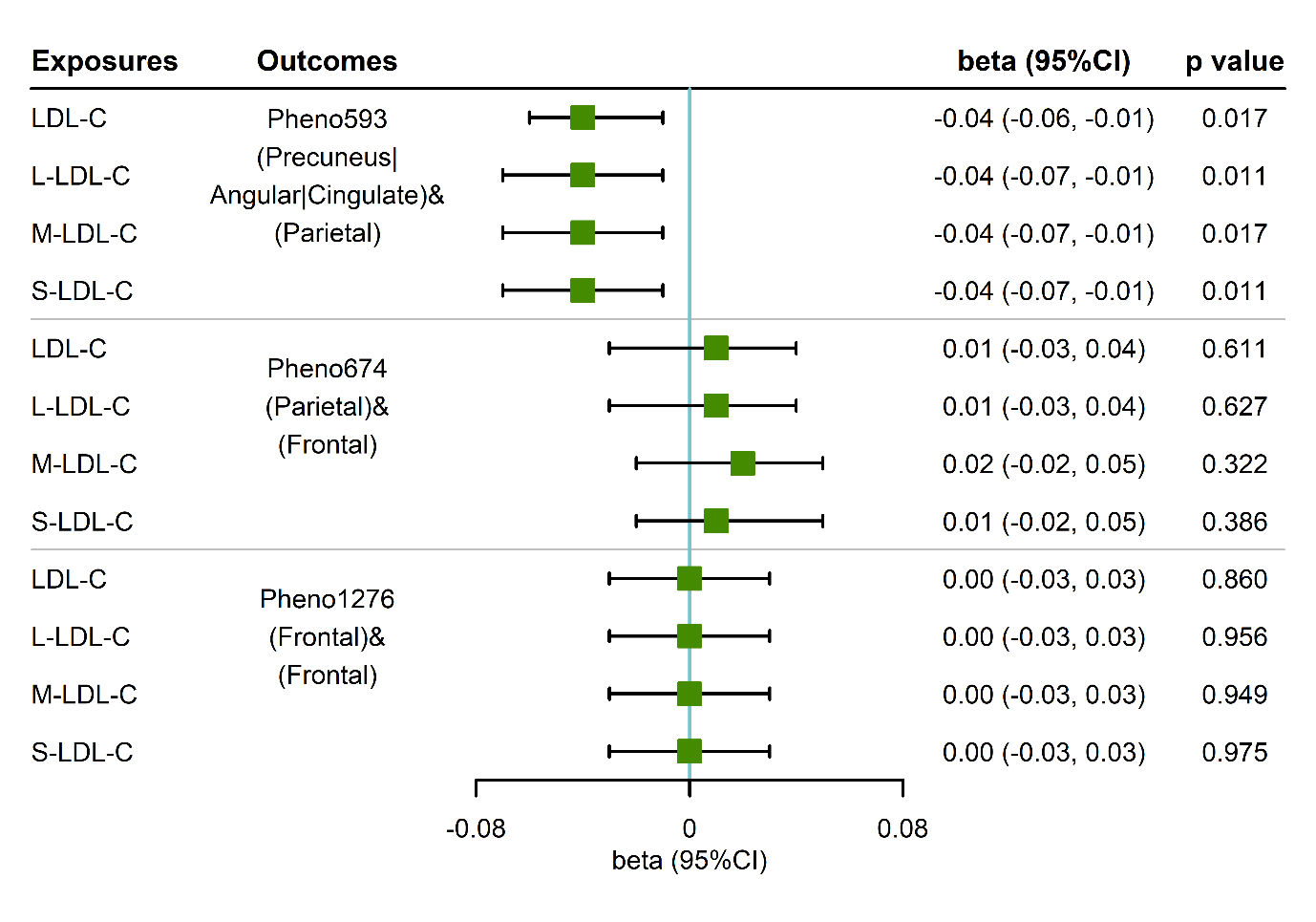
 **Supplementary Figure 9** MR associations of genetically predicted LDL-C and its subfractions with three significant imaging phenotypes that have a causal effect on post-stroke cognitive recovery; results derived from multiplicative random-effects inverse variance weighted analyses.
